# Phonocardiography based pulse wave velocity system for non-occlusive assessment of arterial stiffness

**DOI:** 10.3389/fcvm.2025.1481836

**Published:** 2025-01-23

**Authors:** T. Corina Margain, Emily Powell, Alexandra Clark, Adam Bush

**Affiliations:** ^1^ Biomedical Engineering Department, Translational Cardiovascular Imaging Group, The University of Texas at Austin, Austin, TX, United States; ^2^ Mechanisms Underlying Neurocognitive Aging Laboratory, Department of Psychology, The University of Texas at Austin, Austin, TX, United States

**Keywords:** arterial stiffness, pulse wave velocity, phonocardiography, plethysmography, cardiovascular health

## Abstract

Arterial stiffness is strongly associated with vascular aging and pathology and can be assessed in many ways. Existing devices for measuring central arterial stiffness, such as carotid-femoral pulse wave velocity (PWV), are limited by high costs and the need for specialized expertise, limiting widespread clinical adoption. This study introduces a semi- and non-occlusive PWV measurement system using phonocardiography (PCG) and plethysmography (PPG) and a single femoral pressure cuff, aiming to address these limitations. We conducted a study comparing a semi-occlusive (carotid-femoral PWV) and a non-occlusive (carotid-toe PWV) PCG-based PWV measurements across a cohort of 63 volunteers, as compared to literature reference PWV values. Results demonstrated strong correlations between our PCG-based PWV measures (PWV_carotid−femoral_: 8.42 ± 3.99 m/s vs. PWV_carotid−toe_: 10.62 ± 3.86 m/s) with age as a significant predictor (PWV_carotid−femoral_: *r*^2^ = 0.45; PWV_carotid−toe_: *r*^2^ = 0.28, *p* < 0.05). Ultrasound measured distensibility assessments confirmed the reliability of our PCG approach in reflecting central arterial stiffness dynamics, particularly at the aortic level. Test–retest reliability analyses yielded high intraclass correlation coefficients (0.75 ≤ ICC ≤ 90), indicating robust repeatability of our method. This study highlights the feasibility and accuracy of our low-cost, semi and non-occlusive PWV measurement systems to enhance accessibility in arterial stiffness assessments, potentially easing cardiovascular risk stratification.

## 1 Introduction

### 1.1 Physiology of arterial stiffness

Arterial stiffness refers to the reduced ability of arterial walls to deform under pressure, a process influenced by aging and pathologic conditions like hypertension, atherosclerosis, and nephropathy (1–5). Many studies have shown that increased arterial stiffness is associated with poor outcomes and several have proposed and used arterial stiffening as a predictive biomarker of disease (6–11).

Arterial stiffening can occur throughout the arterial tree and can be measured in many ways. Measurement techniques can be separated into those that measure a small, localized site and those that provide spatial averaged measures across long arterial segments (12). The most widely used metrics of arterial stiffness are those that measure arterial stiffening over a vessel length by quantifying the pulse wave velocity (PWV).

### 1.2 Pulse wave velocity

The PWV represents the speed of wave propagation along a vessel of interest and can be related to the mechanical stiffness of the flowing medium, in this case the blood and arterial wall, by the Moens–Korteweg equation (Equation 1) (13–15) where PWV is the pulse wave velocity, *ρ* is the blood density, $E_{\mathrm{inc}}$ is the vessel wall incremental elastic modulus also interpreted as the wall's distensibility (ΔPR/ΔR) (16), ΔP is change in blood pressure, ℎ is the wall thickness of the vessel, *R* is vessel radius.


(1) $$\mathrm{PWV}=\sqrt{\frac{E_{\mathrm{inc}}h}{2R\rho }}$$

In early age, the highest large artery wall distensibility is found in the proximal aorta and decreases distally across the arterial tree. Aortic stiffness is strongly linked to various systemic vascular diseases (1); however, it can only be accurately assessed through direct measurement of aortic or central PWV, which typically requires invasive techniques or costly imaging modalities (MRI) that are not widely accessible (17, 18).

Indirect central PWV measures, such as carotid-femoral PWV (PWVcf), have emerged as feasible alternatives. PWVcf, which measures pulse speed between the carotid and femoral arteries, closely mirrors direct techniques and is predictive of cardiovascular outcomes, making it the clinical standard for assessing arterial stiffness (19).

### 1.3 Distensibility

Arterial stiffness can also be measured locally by the distensibility of blood vessels, which refers to their ability to stretch in response to a change in pressure (20). Both aortic and carotid artery distensibility are commonly used as non-invasive methods to assess arterial stiffness, as determined by Equation 1. However, few studies have compared direct measurements of local distensibility and systemic assessments like carotid-femoral PWV (PWVcf) (21, 22). Furthermore, the extent to which distensibility measurements at different sites align with systemic arterial stiffness assessments like PWV is seldom addressed.

### 1.4 Existing techniques and limitations of central PWV

PWVcf is typically measured using tonometry at the carotid and femoral arteries or a pressure cuff on the thigh. Tonometry detects pressure changes by flattening the artery, while cuff measurements fully occlude the artery to measure pressure changes (23–25). Other forms of indirect central PWV such as brachial-ankle (26), finger-toe (27) and heart-wrist (28) do not accurately reflect central PWV (29).

Commercial devices like SphygmoCor and Complior are validated but expensive and inaccessible for routine use, particularly in resource-limited setting (30–34). Furthermore, both cuff and tonometry methods can cause discomfort and distort readings by altering pulse wave travel time (35, 36). These limitations have restricted the use of PWV equipment in clinical settings (37).

To overcome the limitations of existing central PWV approaches, plethysmography (PPG) and phonocardiography (PCG) based PWV measurements have been introduced. PPG has been used to measure PWV, but it has been applied to stiffness measurements from arterial extremities such as fingers and toes which are not representative of central arterial stiffness (38–41). Alternatively, PCG methods have been used to measure heart sounds captured at the ear, to replace the ECG as the heart-trigger reference, and at the femoral and carotid arteries for PWVcf measurements (42–44). However, these studies were limited by small sample sizes, narrow age ranges, and not compared to reference PWV values.

Therefore, the goal of this work was to develop an easy to use, non-occlusive, low-cost alternative to traditional PWV assays using the following approach.

## 2 Materials and equipment

Our PWV measurement system consisted of the following: A Biopac (Biopac Systems, Goleta, CA) MP36 DAQ system that acquired synchronized biosignals, including electrocardiogram (SS2LB) and leads, PCG (SS17LA) at the carotid, PPG (SS4LA) at the toe, and pressure cuff (SS19LB) data on the thigh (34).

Additional equipment included an Omron Digital Blood Pressure monitor (OMRON Corporation, Shiokoji Horikawa, Kyoto), a handheld point of care ultrasound system (POCUS) Butterfly IQ+ (Butterfly Network, Inc, Burlington, MA), and Apple smartphone (Apple Inc. Cupertino, CA) for image derived distensibility measurements. Software tools for signal processing and statistical analysis included Matlab (Mathworks, Natick, MA) and JMP 16 (JMP Statistical Discovery LLC, Cary, NC).

## 3 Methods

### 3.1 Study protocol and pre-test instructions

All studies were conducted following the University of Texas at Austin Institutional Review Board approval, ensuring compliance with ethical standards for human research. Informed consent was obtained from all participants before enrollment, and subjects were fully briefed on the study objectives, procedures, and their right to withdraw at any time without penalty.

We enrolled a cohort of 63 volunteers (78% female), spanning an age range of 18–83 years, with a mean age of 51 ± 21 years. Subjects were recruited voluntarily during the recruitment phase and were not compensated for participation. To protect participant privacy, all data were de-identified using acrostic subject IDs. The de-identified data were securely stored and accessible only to authorized investigators. Additionally, data handling adhered to institutional and federal guidelines to ensure confidentiality and mitigate risks.

Subjects were prepared by resting in a supine position for at least 5 min before the study (45). Data on age, height, weight, gender, and blood pressure (BP) were collected for each subject (Table 1). The study was completed in less than 10 min.

**Table 1:** Demographic overview (mean ± sd) of study volunteers (*n* = 63).

Age (years) (*n* = 63)	51 ± 21
Sex (Male/Female)	49/14
Height (cm)	160.27 ± 15.84
Weight (kg)	73.11 ± 20.75
Systolic Blood Pressure (mmHg)	122.36 ± 16.86
Diastolic Blood Pressure (mmHg)	75.86 ± 9.98
Mean Arterial Pressure (MAP, mmHg)	91.37 ± 11.58
PWVct (m/s)	10.67 ± 3.87
PWVcf (m/s)	8.42 ± 4.03

The main study consisted of measuring PWV from probes/cuffs positioned at the right carotid artery, at the right toe and on the upper right thigh. The cohort was divided into two substudies:

Substudy #1: Assessment of aortic and carotid artery distensibility
Substudy #2: Evaluation of the reproducibility and repeatability of the PCG-based PWV method.

**Figure 1:**
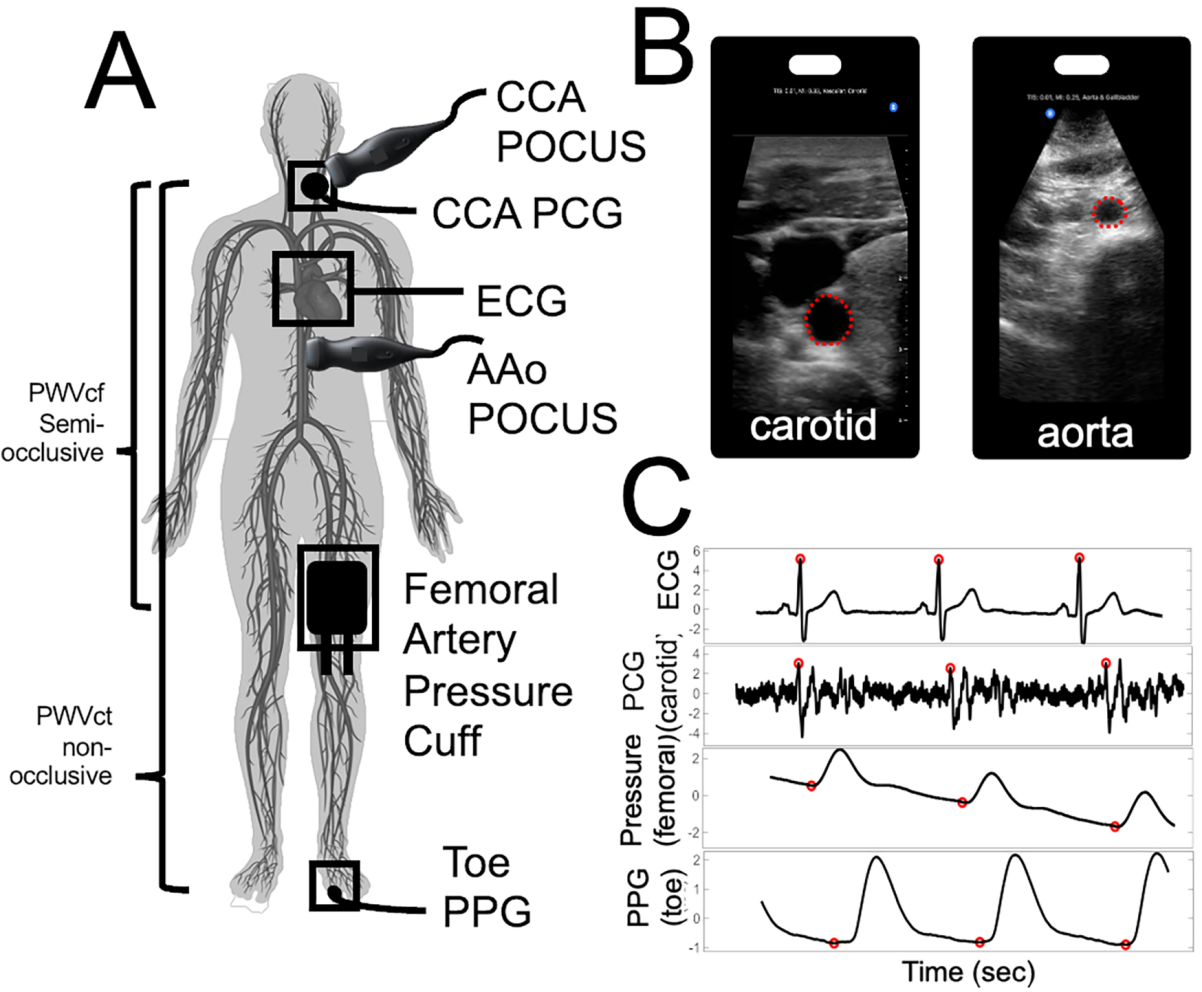
Pictorial representation of our stiffness assay set-up to capture central arterial stiffness. **(A)** Location of the probes for PWV and distensibility index evaluation. Highlighting our semi-occlusive carotid-femoral PWVcf and our non-occlusive carotid-toe PWVct. **(B)** Point-of-Care Ultrasound (POCUS) images and ROI selected for carotid artery (left) and abdominal aorta (right) **(C)** BIOPAC biosignals and automated foot finding algorithm with respect to ECG R-peaks.

Figure 1 provides a pictorial representation of the setup and acquired biosignals.

### 3.2 Quality control and inclusion/exclusion criteria

We applied quality control measures and inclusion/exclusion criteria to ensure the reliability and validity of our findings. Instances where the Biopac data acquisition comprised fewer than ten cardiac cycles or where biosignals were compromised by artifacts such as abnormal ECG QRS complexes, movement, or speech during examination were excluded. Additionally, data from participants who showed intolerance to the inflated femoral cuff were excluded. POCUS data with suboptimal spatial resolution or motion artifacts were also excluded.

Subjects with hypertension, defined as systolic blood pressure exceeding 135/80 mmHg for individuals under 65 years-old and 150/80 mmHg for those over 65 years old, were also excluded (46, 47). A total of ten participants with hypertension were excluded.

Beyond hypertension, we did not implement other exclusion criteria to exclude arterial obstructions or explicitly screen for subjects with cardiac valvular disease or murmurs.

### 3.3 Pulse wave velocity (PWV) measurements

All measurement signals were recorded and synchronized using a Student Biopac MP 36 system and Biopac transducers with sampling rate of 2,000 Hz. Pulse wave velocity was calculated as (31):

$$\mathrm{PWV}(m/s)=\frac{\mathrm{distance}\;}{\mathrm{pulse}\;\mathrm{delay}\;\mathrm{time}}$$

In this study, we employed two PWV measurement approaches: semi-occlusive and non-occlusive PWV assessments. Semi-occlusive measurements involve partial restriction of blood flow using a pressure cuff (e.g., carotid-femoral PWV), whereas non-occlusive measurements do not involve any occlusion (e.g., carotid-toe PWV). This distinction underpins the methods used in the following PWVct and PWVcf sections.

#### 3.3.1 Carotid-toe pulse wave velocity (PWVct)

Carotid-Toe Pulse Wave Velocity (PWVct) measurements were performed with a Biopac PCG (SS17LA) at the carotid and PPG (SS4LA) at the big toe. These PWVct measurements were therefore entirely non-occlusive (Figure 1A).

For analysis, the S1 heart sound on the carotid was detected using an automated peak-detection algorithm to find the first largest change in the signal's amplitude (42, 43). The foot of the PPG signal at the toe was detected via a foot-detection algorithm described elsewhere (45). The difference between the average of at least five detected PCG and PPG signals and the peak R-wave of the ECG signal was used to determine the pulse delay time for each measurement site (Figure 1C). The straight-line distance, between the carotid and toe measurement site was divided by the pulse delay time to calculate PWVct.

#### 3.3.2 Carotid-femoral pulse wave velocity (PWVcf)

Carotid-Femoral Pulse Wave Velocity measurements were performed with a Biopac PCG (SS17LA) at the carotid and Biopac pressure cuff (SS19LB). These PWVcf measurements were therefore semi-occlusive due to use of a thigh cuff.

For acquisition, the carotid PCG heart sounds were recorded, while the blood pressure cuff was briefly inflated to 10 mmHg above the participant's systolic pressure, which was estimated based on the brachial blood pressure while the participant was in a supine position. This estimation assumes a similar pressure gradient between the brachial and femoral arteries in this position. The brief inflation of the cuff was enough to temporarily restrict blood flow without fully occluding the femoral artery, allowing us to record the femoral pulse wave (Figure 1C). Approximately 10 cardiac cycles were recorded before the cuff was deflated.

For analysis, the foot of the pressure pulse was detected with the same foot-detection algorithm from the PPG used in PWVct measurements, and the time delay between the ECG R-wave and the pressure pulse was calculated. The average of the pulse delay times across a minimum of five cardiac cycles was used for analysis. Finally, the straight-line distance between the carotid and femoral measurement sites was divided by the pulse transit time and multiplied by 0.8 to calculate PWVcf (31).

#### 3.3.3 Comparison with reference PWVcf data

To compare our PCG-based PWV with reference PWVcf values obtained using validated devices [Sphygmocor (44–49), Vicorder (48–51), Pulse Pen (52) and Complior (53, 54)], we utilized data from studies that reported PWVcf for 1911 healthy subjects (ages 18–91, mean age 49 ± 18). These studies were selected because they used a validated device and included a healthy population with a similar age range to our study. We digitized the image data from scatter plots in these studies using an online tool (55).

All processing was performed using in-house, automated Matlab scripts created for this purpose.

### 3.4 Substudy 1: distensibility Index measurements

To determine the agreement between separate measures of arterial stiffness between modalities, we also assessed carotid and abdominal aortic distensibility index in a subset of the study population. Where instead of calculating distensibility (ΔArea) (Area_diastolic_ × pulse pressure) we calculated the distensibility index to provide a stiffness metric that is independent of changes in blood pressure, and we used the formula (22, 56, 57):

$$\mathrm{Distensibility}\;\mathrm{Index}=\frac{1}{\mathrm{stiffness}\;\mathrm{index}(\beta )}=\frac{\mathrm{strain}}{\ln\;\left[\frac{\mathrm{SBP}}{\mathrm{DBP}}\right]}$$

where SBP and DBP (systolic and diastolic blood pressure) was taken at the arm and strain was calculated from the ratio of change in systolic cross-sectional area over diastolic cross-sectional area (7, 57–59). Ultrasound exams were performed on the same day and during the same session as PWVcf and PWVct measurements.

Ultrasound examinations were performed in supine position using a Butterfly IQ+ point of care ultrasound system (POCUS) in B-mode carotid and abdominal settings (60). Ten to twenty second recordings of the right common carotid and abdominal aorta in a transverse plane were measured and processed using manual edge detection (Figure 1B). Systolic and diastolic areas were calculated and the average across at least 3 cardiac cycles was used to determine the abdominal aorta (AAo Dist) and common carotid artery (CCA Dist) distensibility index (58, 59).

### 3.5 Substudy 2: test–retest

We evaluated the reproducibility of our PWV assay in ten participants. PWVct and PWVcf were measured twice, with a 2-min interval (intrasession) and after 7 days (intersession) (61–63). Intraclass correlation coefficients (ICCs) were calculated to assess reliability (*p* < 0.05).

To determine the influence of occlusion type and confirm that the PCG time-delay corresponds to a pulse detected from the local vessel wall contraction, rather than sound traveling from the heart to the PCG probe at various anatomical locations, we compared PCG derived time-to-foot (TTF) delays at the heart, carotid, and femoral arteries in 5 subjects. The PCG sensor was placed directly on the femoral pulse near the groin. Femoral pulse detection techniques included: cuff occlusion-derived pressure signal, direct auscultation with an unoccluded PCG, and auscultation with PCG under distal cuff occlusion. The peak R-wave ECG signal served as the reference, and PCG sensors measured pulse travel times from the heart and carotid sites.

We calculated the mean differences in TTF delays from the ECG R-peak to signals detected at the heart, carotid, and femoral arteries (Supplemental Material).

### 3.6 Statistical methods

All statistical analyses were performed using JMP 2022, with *α* levels set to 0.05.

For the PCG PWVcf and PWVct measurements, statistical tests included linear regressions, as well as univariate followed by multivariate analyses to examine the relationship between our PWV methods, age, height, weight, gender, and brachial blood pressure. A Bland–Altman analysis was performed to evaluate the agreement and bias between the non-occlusive (PWVct) and semi-occlusive (PWVcf) PCG-based methods.

To compare our PCG-based PWVcf to reference PWVcf measurements (Sphygmocor, Vicorder, Pulse Pen and Complior), multiple linear regression models were applied to the historical data with the PCG-based PWVcf and PWVct. Interaction terms were included in the models to evaluate whether the relationship between PWV and age differed across PCG-based and validated devices. Additionally, since the sample size of the historical data was larger than our study's sample size, we normalized the reference data by randomly subsampling the historical data to match our study's sample size (*n* = 63) and we performed the same linear regression models on the re-sampled data. Random sampling without replacement ensured equal, unbiased sample sizes, and averages of *R*^2^ and *p*-values were computed from 100 iterations (64). Agreement between each reference device was also assessed using this approach.

For the distensibility index study, univariate analyses and linear regressions were conducted to assess the relationship between distensibility index, PWV, and age. Results were considered statistically significant at *p* < 0.05.

For the test–retest data, test–retest reliability was assessed using a Test of Equivalence (TOST) with a difference threshold of <1.5 m/s. A Tukey test was used for pairwise comparison of TTF means to determine statistical differences, with significance set at *p* < 0.05 (31) (Supplementary Figure 1).

## 4 Results

### 4.1 General study results

**Figure 2:**
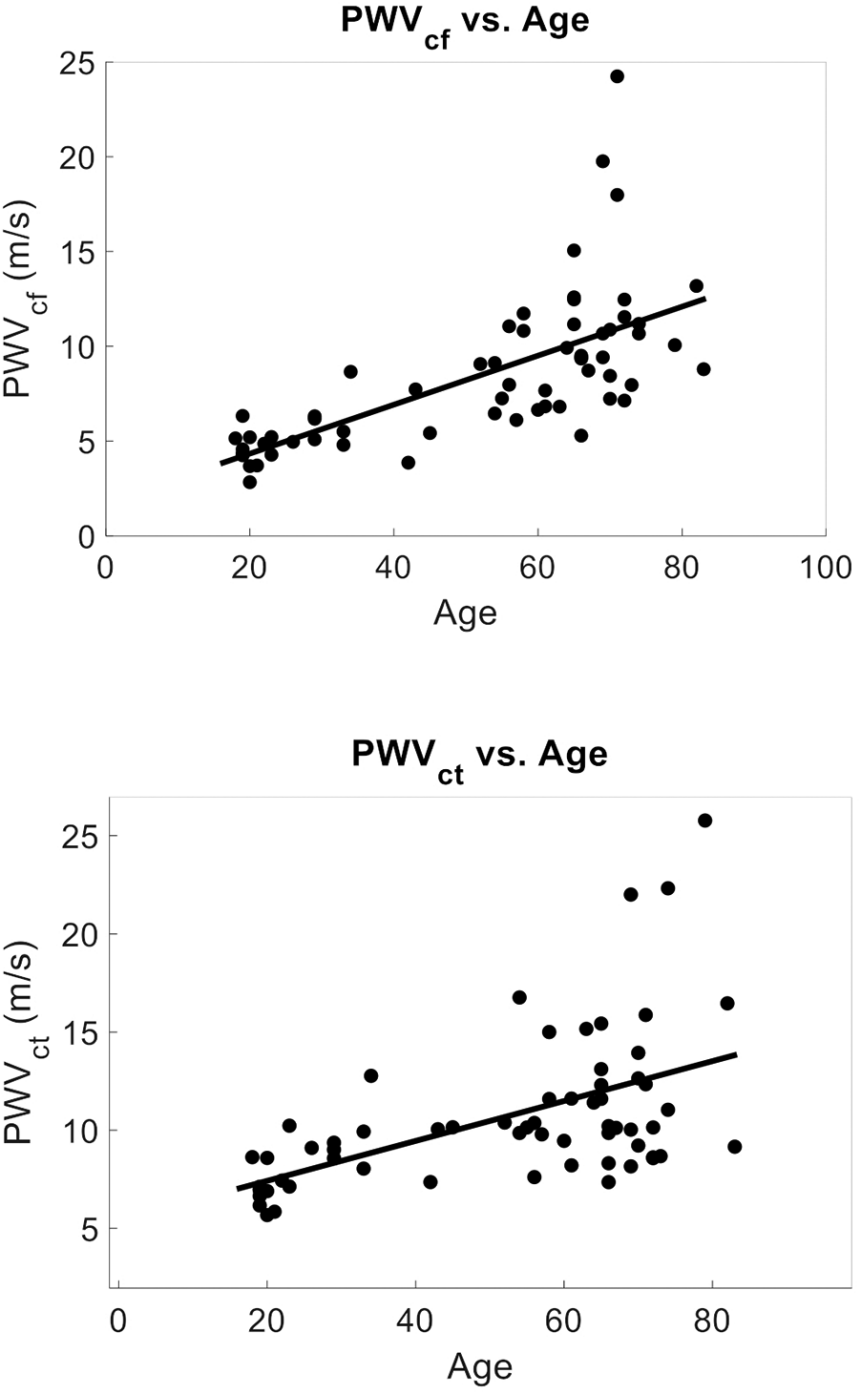
PCG-based PWV vs. age (Mean age = 51 ± 21): (top) PWVcf vs. age. *r*^2^ = 0.45, *p* < 0.001, *n* = 63. Mean PWVcf = 8.42 ± 3.99 m/s. (bottom) PWVct vs. age *r*^2^ = 0.28, *p* < 0.0001, *n* = 63. Mean PWVct = 10.62 ± 3.86 m/s. PWV was strongly predicted by age on univariate and multivariate analysis in agreement with literature.

Among the 63 volunteers, the mean PWVcf was 8.42 ± 3.99 m/s, while PWVct was 10.62 ± 3.86 m/s. Univariate analyses revealed significant associations between age and PWV (PWVcf *r*^2^ = 0.45 and PWVct *r*^2^ = 0.28, *p* < 0.0001) (Figure 2). Other parameters associated with PWVcf and PWVct on univariate analysis included mean arterial pressure (PWVcf *r*^2^ = 0.13, *p* = 0.003 and PWVct *r*^2^ = 0.10, *p* = 0.013), systolic blood pressure (PWVcf *r*^2^ = 0.14, *p* = 0.003 and PWVct *r*^2^ = 0.09, *p* = 0.016), and diastolic blood pressure (PWVcf *r*^2^ = 0.10, *p* = 0.01 and PWVct *r*^2^ = 0.08 *p* = 0.023).

Upon multivariate analyses age was the principal predictor for both PWVcf (*F* Ratio = 182.66, *r*^2^ = 0.62, *p* = 3.4 × 10^−25^) and PWVct (*F* Ratio = 89.90, *r*^2^ = 0.45, *p* = 5.6 × 10^16^).

**Figure 3:**
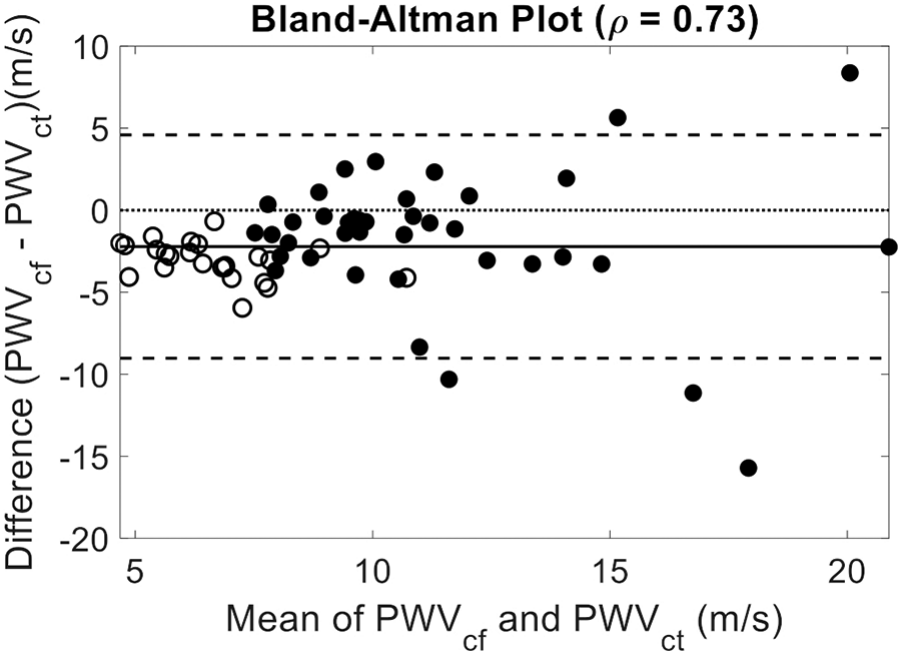
Bland–Altman comparison of PWVct and PWVct. Mean Bias: −2.21 (*p* < 0.0001); 95% Confidence Interval: [−3.08, −1.36], *ρ* = 0.73, Limits of Agreement: [−9.01, 4.60]. Closed Circles for subjects older than fifty years old. Showing good agreement between non-occlusive and semi-occlusive PWV methods. The observed bias likely reflects the divergence in central and peripheral arterial stiffness, with central PWV increasing more significantly with age than peripheral PWV.

A Bland–Altman analysis comparing PCG-based semi-occlusive PWVcf and non-occlusive PWVct methods demonstrated strong agreement (*ρ* = 0.73), with age exerting a notable influence on the observed bias [mean Bias = −2.21 ± 3.47 m/s, *p* < 0.0001, Limits of agreement (−9.01, 4.60)], particularly among older subjects (>50 years old) (Figure 3). (Bias for <50 subjects = −2.86 ± 2.42 m/s, *p* < 0.0001, *ρ* = 0.65, and bias for >50 −1.69 ± 4.26 m/s, *p* = 0.02, *ρ* = 0.48.).

#### 4.1.1 PCG-based PWVcf vs. reference PWVcf devices

**Figure 4:**
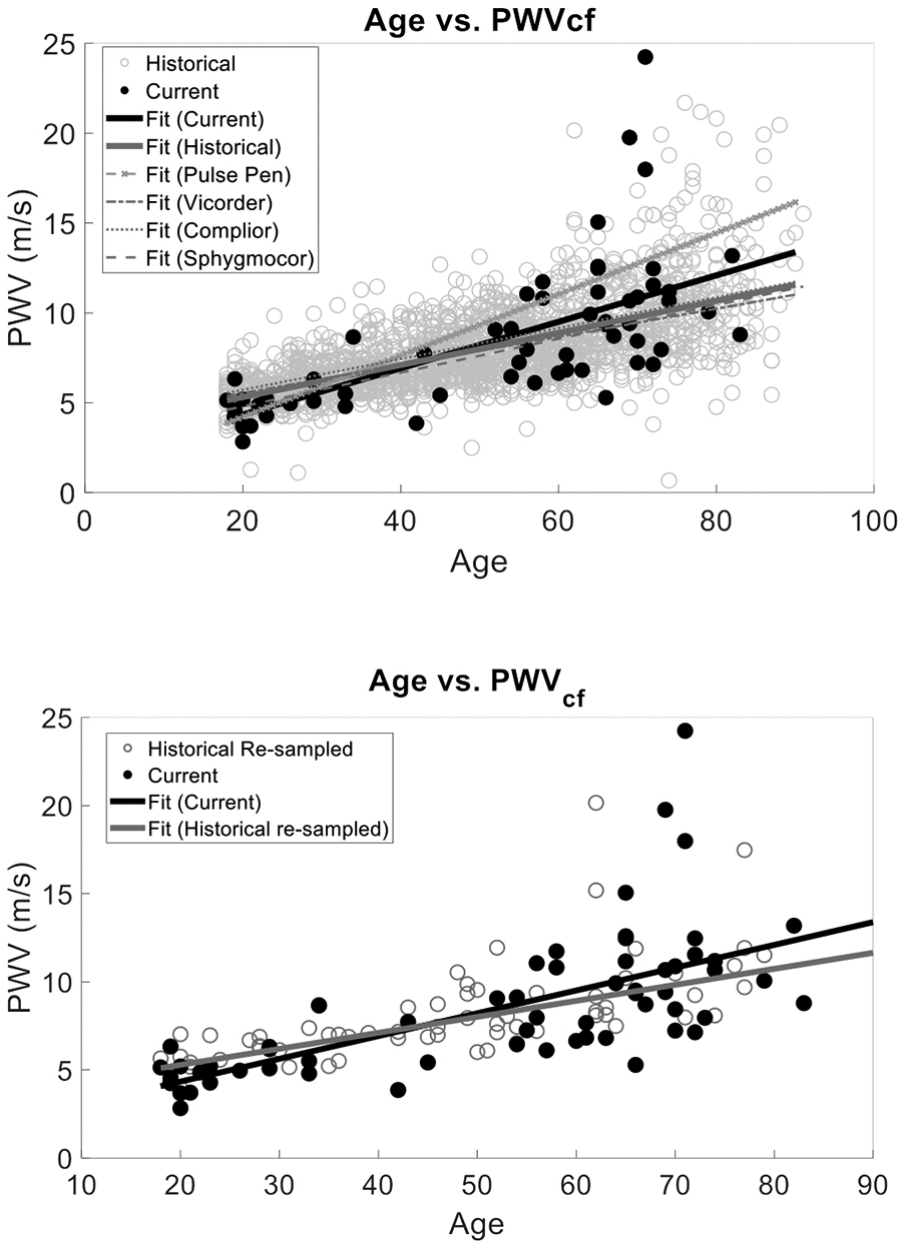
PWV vs. Age validation against data obtained from previous studies that utilized a SphygmoCor, Complior, Pulse Pen, or Vicorder device for PWVcf. Our current method and the historical data have the same relationship between age and PWV (*p* > 0.05) with no statistically significant interaction between slope and current vs. historical measurement type (top). Similarly, (bottom) we compared our semi-occlusive PWVcf to a resampled historical PWVcf data that used the same validated devices (SphygmoCor, Complior, Pulse Pen, or Vicorder) and found that the slopes of the linear fits were the same (*p* > 0.05).

To provide evidence of the agreement of our approach to four historical SphygmorCor, Complior, Pulse Pen, and Vicorder data, (mean PWVcf of 7.92 ± 2.37 m/s) (48–54, 65–70), we performed multiple linear regressions of age vs. PWV with interaction correction for current or historical measures. The models included age, study type (current vs. historical), and their interaction as predictors. Although PWVcf current and PWVcf historical were correlated with age (*r*^2^ = 0.44, *p* < 0.0001), there was no statistical difference between study type (*r*^2^ = 0.46, *p* = 0.42). However, the interaction between age and study type was statistically significant (*p* = 0.0005) (Figure 4).

To determine the unbiased effect size of the interaction of study type and age, we matched the sample sizes between our PCG PWVcf data (*n* = 63) and historical data by randomly re-sampling the historical data. We performed resampled regressions as described above for 100 iterations. The average *p*-values and combined *r*^2^ were calculated and showed that age remained strongly correlated with PWVcf (*r*^2^ = 0.44 ± 0.02, *p* < 0.0001). The study type (*p* = 0.58) and the interaction between study and age (*r*^2^ = 0.46 ± 0.03, *p* = 0.12) were not statistically significant in this analysis (Figure 4).

##### 4.1.1.1 Device specific comparisons

**Figure 5:**
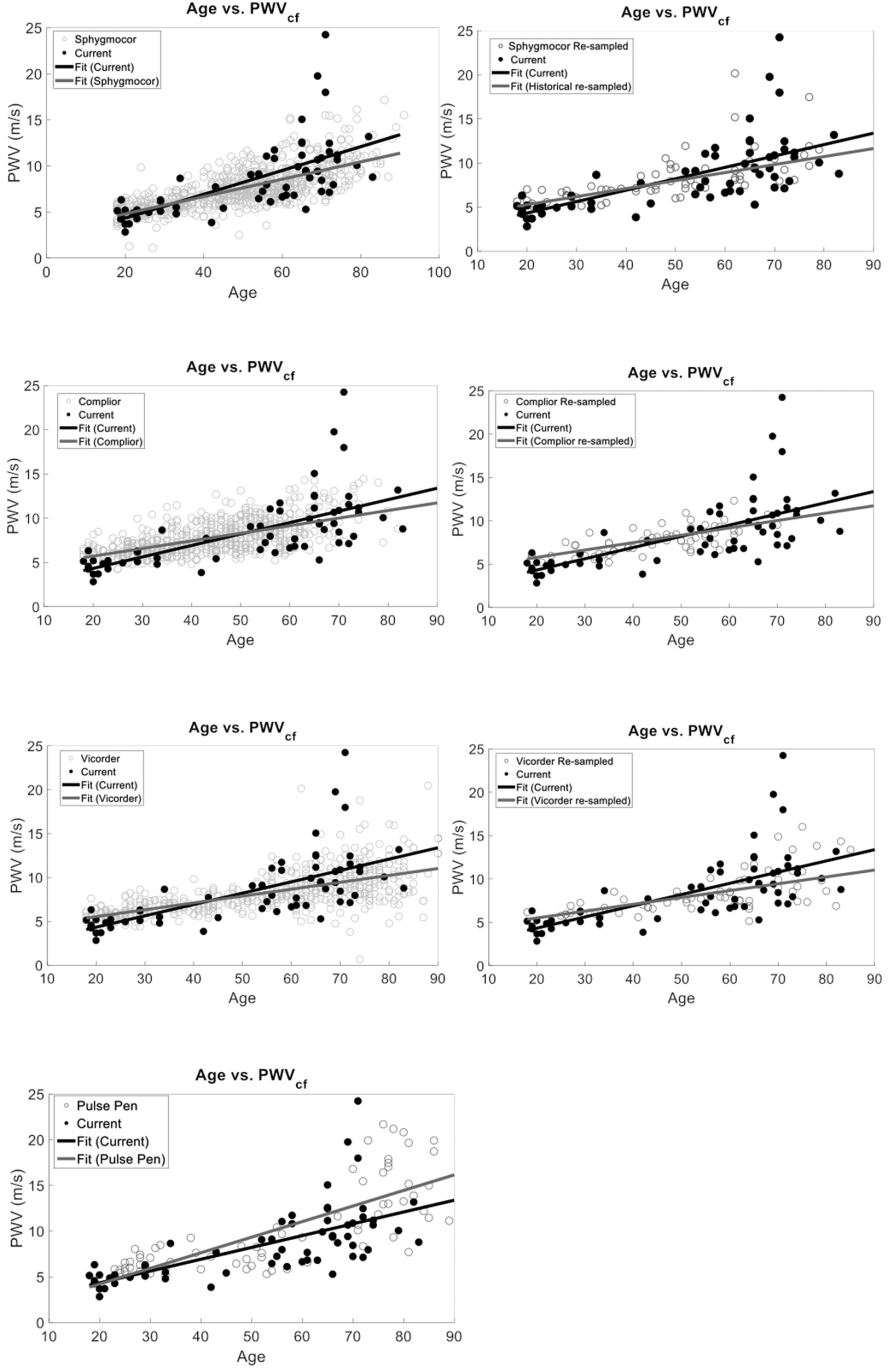
Device-specific comparisons of PCG-based PWVcf against historical reference devices (SphygmoCor, Vicorder, Pulse Pen, and Complior). Panels show full dataset comparisons (right) alongside resampled datasets (left, except for Pulse Pen). Significant differences between our device were observed for SphygmoCor (*p* = 0.01), Vicorder (*p* = 0.04), and Pulse Pen (*p* = 0.02) in the full data but became non-significant after resampling (*p* > 0.05), except for Pulse Pen which was not resampled. Interaction effects between age and study type were significant for Vicorder (*p* = 0.35) in the resampled analysis but not for SphygmoCor (*p* = 0.15), Complior (*p* = 0.67), or Pulse Pen (*p* = 0.10).

Device-specific comparisons to our PCG-based PWVcf device showed a significant difference in PWVcf for SphygmoCor (*p* = 0.01), Vicorder (*p* = 0.04) and Pulse Pen (*p* = 0.02), but not for Complior (*p* = 0.16). The interaction between device and age was also statistically significant for all devices (*p* < 0.05) except for Pulse Pen (*p* = 0.10) (Figure 5).

We also re-sampled each device data set to normalize to our study sample size. Since the Pulse Pen had a sample size comparable to ours (*n* = 70) it was not re-sampled for the analysis. Device-specific comparisons using resampled datasets showed no significant difference in PWVcf between our PCG-based values and SphygmoCor (*p* = 0.19), Complior (*p* = 0.67), or Vicorder (*p* = 0.35). For the Pulse Pen (not resampled), a significant difference in PWVcf was observed (*p* = 0.02). The interaction between device and age was significant for Vicorder (*r*^2^ = 0.43, *p* = 0.04), while it was not significant for Complior (*r* ^2^ = 0.41, *p* = 0.15), SphygmoCor (*r*^2^ = 0.51, *p* = 0.15), Pulse Pen (*r*^2^ = 0.57, *p* = 0.10) (Figure 5).

#### 4.1.2 PCG-based PWVct vs. reference PWVcf devices

**Figure 6:**
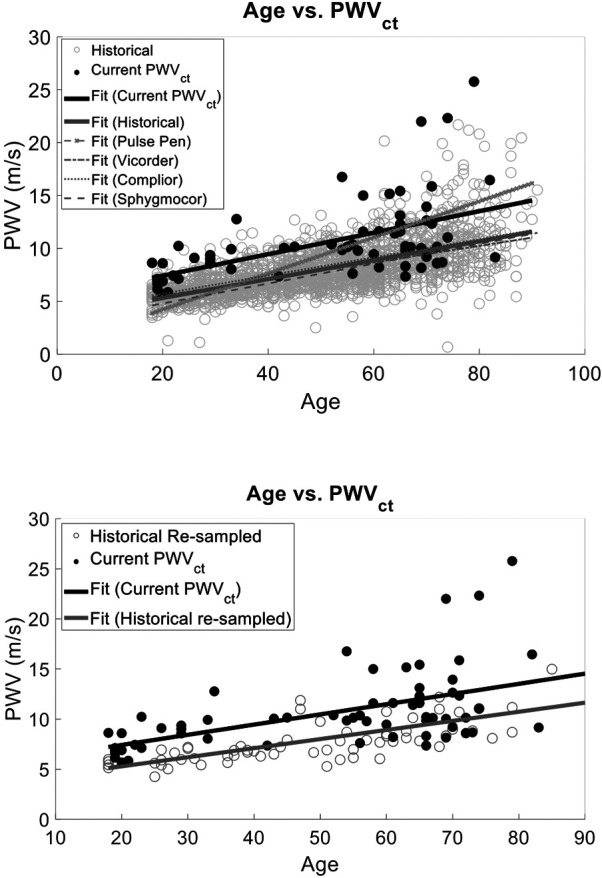
We compared our cuffless PWVct to a the historical PWVcf data that used validated devices (sphygmoCor, complior, pulse Pen, or vicorder) and found that the slopes of the linear fits were the same (*p* > 0.05). However, there was a statistically significant interaction with intercept (F Ratio = 76.72, *p* < 0.001) demonstrating the bias between historical PWV measurements and PWVct of this study. This suggests the age vs. PWV relationship is reliably captured with our two low-cost PWV methods. However, PWVct will result in population wide biased measurements.

We also compared the PCG-based PWVct and PWVcf historical and there was a correlation with age (*r*^2^ = 0.42, *p* < 0.001) and a statistically significant difference between study type (*p* < 0.001) (Figure 6). However, the interaction between age and study type was not significant (*r*^2^ = 0.45, *p* = 0.29).

Similarly to the analysis for PWVcf, we normalized the historical data to match our PWVct sample size as described in section 4.1.1. The correlation between age and study type remained significant (*r*^2^ = 0.31 ± 0.02, *p* < 0.0001), and the interaction term between age and study type was also not significant (*r*^2^ = 0.44 ± 0.04, *p* = 0.56) (Figure 6).

### 4.2 Substudy 1 results: arterial distensibility index

**Figure 7:**
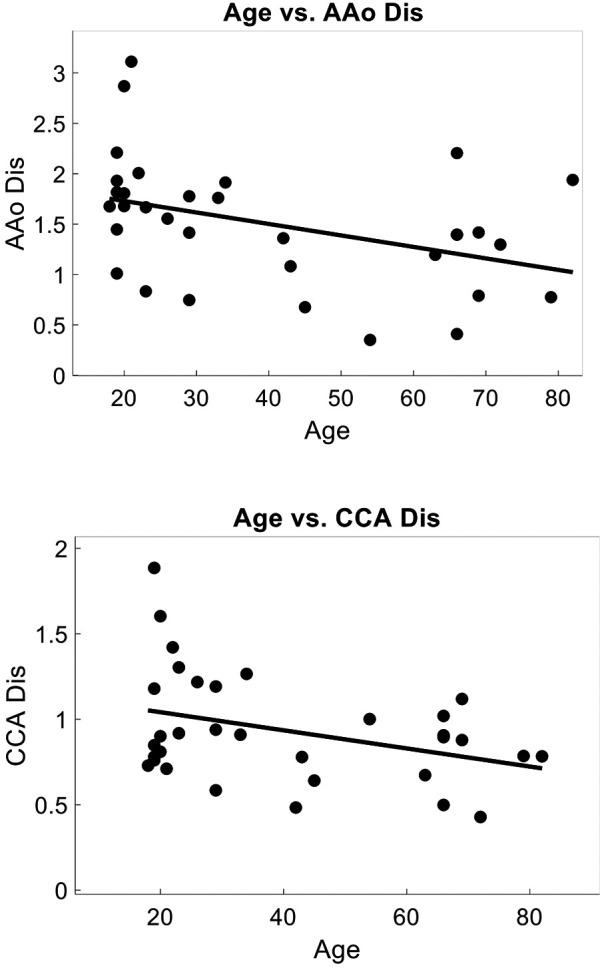
Correlation plots showing relationship between arterial distensibility index decreasing with age. Measured with POCUS at the aortic artery (AAo), correlation coefficient = −0.47, *p* = 0.007, *r*^2^ = 0.22, (top) and at the common carotid artery (CCA), correlation coefficient = −0.36, *p* = 0.045, *r*^2^ = 0.13. (bottom).

**Figure 8:**
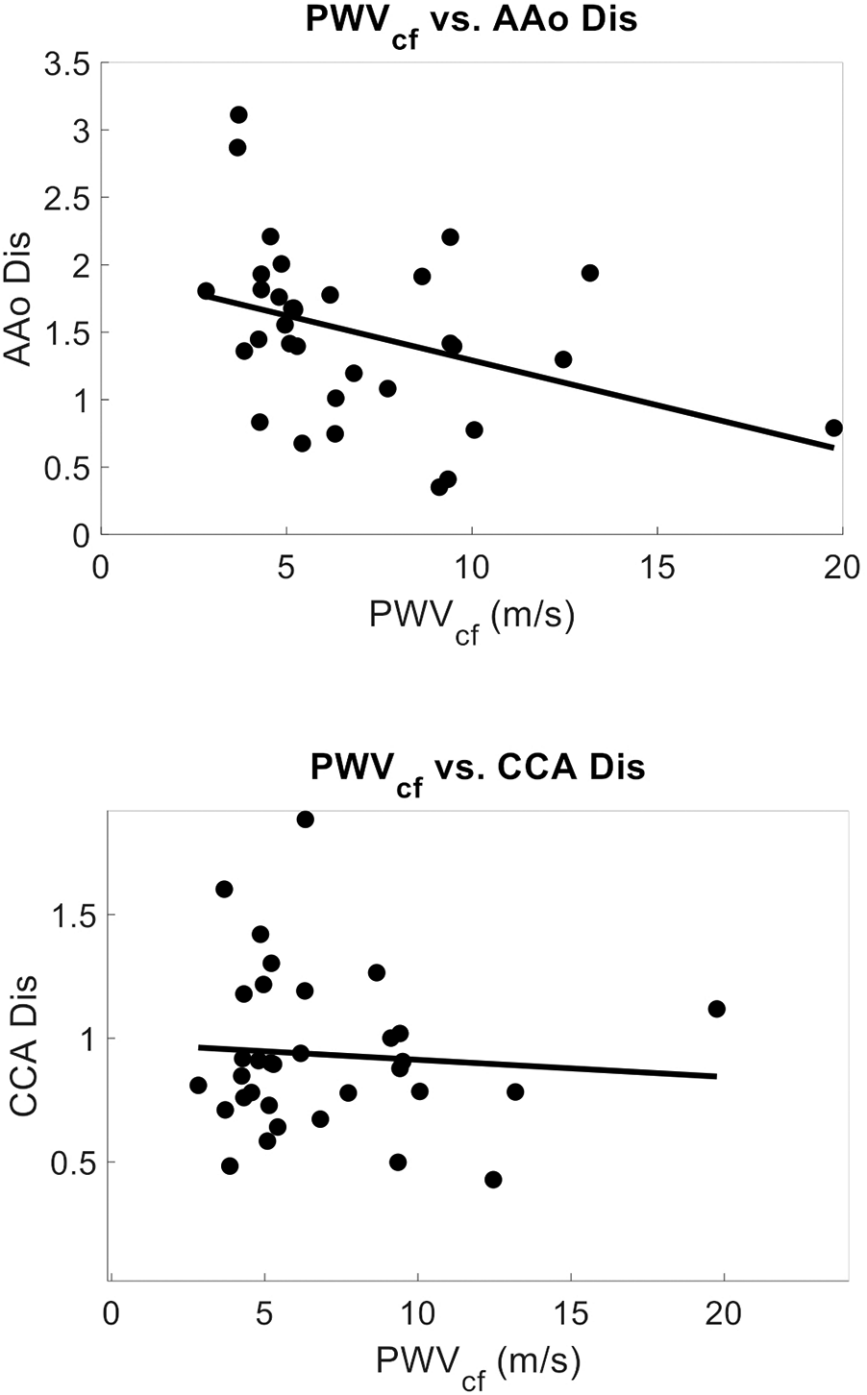
Correlation plots showing relationship between arterial stiffness measured with PWVcf and arterial distensibility index measured with POCUS at the aortic artery (AAo), correlation coefficient = −0.45, *p* = 0.012, *r*^2^ = 0.12(top), and at the common carotid artery (CCA), correlation coefficient = −0.06, *p* = 0.77, *r*^2^ = 0.003 (bottom).

**Figure 9:**
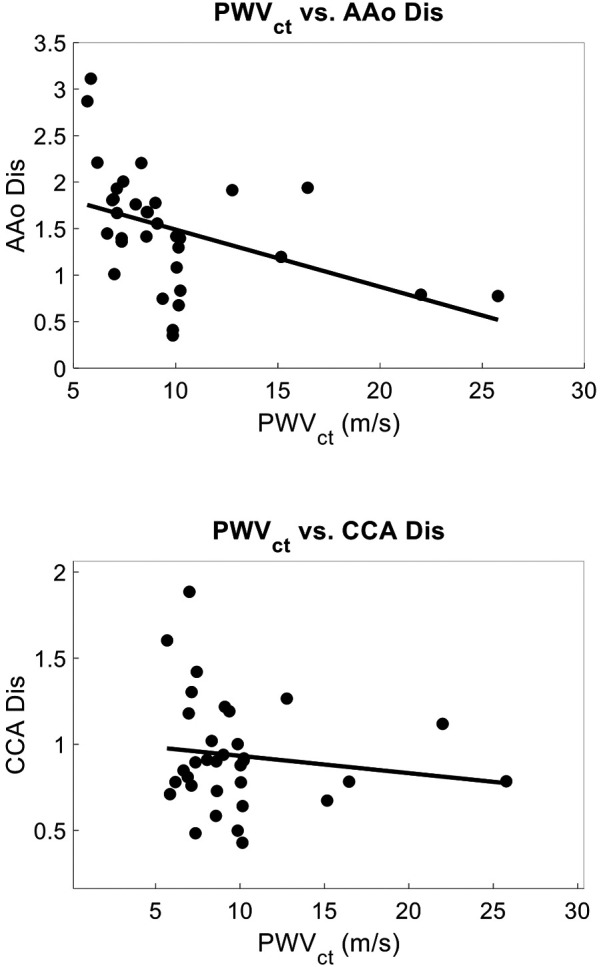
Correlation plots showing relationship between arterial stiffness measured with PWVct and arterial distensibility index measured with POCUS at the aortic artery (AAo) correlation coefficient = −0.50, *p* = 0.003, *r*^2^ = 0.25, (top) and at the common carotid artery (CCA) (bottom), correlation coefficient = −0.11, *p* = 0.56, *r*^2^ = 0.01 indicating that PWVct can detect central arterial stiffness as measured with localized AAo distensibility index, whereas local CCA distensibility index is not representative of central stiffness.

Within a subset of 32 participants (age 39 ± 21, 78% female), the distensibility indices were also calculated. The AAo Dis averaged 1.49 ± 0.63, while the common CCA Dis was 0.94 ± 0.32. Univariate analysis revealed significant negative correlations between age and arterial distensibility index measured at the AAo (*r* = −0.47, *r*^2^ = 0.22, *p* = 0.01), and at the CCA (r = −0.36, *r*^2^ = 0.13, *p* = 0.04) (Figure 7). Our PCG-based PWVcf and AAo Dis (*r* = −0.45, *r*^2^ = 0.20, *p* = 0.01) measurements were correlated, but not with CCA Dis (*r* = −0.06, *r*^2^ = 0.003, *p* = 0.76) (Figure 8). For PWVct, significant correlations were found with AAo Dis (*r* = −0.50, *r*^2^ = 0.25, *p* = 0.003), but not with CCA Dis (*r* = −0.11, *r*^2^ = 0.01, *p* = 0.56) (Figure 9).

Multivariate analysis identified age as the main predictor for both AAo (*F* Ratio = 23.36, *p* = 4.37 × 10^5^, *r*^2^ = 0.21) and CCA (*F* Ratio = 15.93, *p* = 0.0002, *r*^2^ = 0.23) distensibility index. Additionally, sex (*F* Ratio = 14.2, *p* = 0.001, *r*^2^ = 0.40) and weight (*F* Ratio = 5.73, *p* = 0.02, *r*^2^ = 0.50) were found to be significant predictors for AAo distensibility index.

### 4.3 Substudy 2 results: test–retest and PCG time-to-foot validation

To assess the repeatability and reproducibility of our PCG-based PWV we evaluated the intersession and intrasession ICC. Paired *t*-tests showed no statistical significance (*p* > 0.05) between intrasession PWVct (*p* = 0.93) and intersession PWVct (*p* = 0.77), as well as intrasession PWVcf (*p* = 0.99) and intersession PWVcf (*p* = 0.98). Our two PCG-based PWV methods demonstrated good reliability and repeatability (0.75 ≤ ICC ≤ 90) (Table 2) (31, 63, 71). Intraclass correlation coefficients (ICCs) were used to determine statistical significance for test–retest reliability [*p* < 0.05 using a Test of Equivalence (TOST) with a difference threshold <1.5 m/s] (31).

**Table 2:** Test–retest results for intrasession and intersession PWV (*n* = 10). Showing good (0.75 ≤ ICC ≤ 90) reproducibility and repeatability for both PWVcf and PWVct with 95% confidence intervals.

Variable	Day 1–1	Day 1–2	Day 2–1	Day 2–2	Intrasession ICC [95% CI]	Intersession ICC [95% CI]
PWV_cf_ (m/s) ± SD	5.9 ± 1.4	5.8 ± 1.0	5.9 ± 1.2	5.8 ± 1.2	0.79*** [0.31, 0.97]	0.90*** [0.45, 0.98]
PWV_ct_ (m/s) ± SD	9.5 ± 1.5	9.4 ± 1.8	9.6 ± 2.5	9.8 ± 2.9	0.75* [0.15, 0.96]	0.89* [0.34, 0.97]

*n* = 10; *p < 0.05. ***p < 0.0005.

## 5 Discussion

This study demonstrates the feasibility of a low-cost, phonocardiography-based system for assessing central arterial stiffness. By leveraging PCG and PPG sensors, we have developed a method that is low cost and removes occlusive procedures, patient discomfort, and the need for specialized expertise.

### 5.1 PWVcf and PWVct vs. age

Both PWVcf and PWVct showed a strong correlation with age, with multivariate analysis confirming age as the primary predictor for both measures. These findings align with the well-established relationship between aging and arterial stiffness (72). Furthermore, our results demonstrate that PCG-based PWVcf closely mirrors validated devices (SphygmoCor, Vicorder, Pulse Pen, and Complior) in capturing the age-related progression of arterial stiffness, particularly when historical data were resampled to match our study's sample size.

In contrast, PCG-based PWVct was less consistent. While PWVct correlated with age, significant differences between study types and the absence of a significant interaction between age and study type suggest reduced reliability compared to PWVcf. Normalizing sample sizes did not improve the alignment, further indicating that PWVct is a fundamentally different measure than PWVcf and lacks the sensitivity needed for robust comparisons across devices, particularly in population-based studies.

The Bland–Altman analysis highlights a notable bias between PWVcf and PWVct, particularly in older participants (>50 years), where the arterial stiffness gradient between central and peripheral arteries becomes more pronounced. This bias underscores the limitations of using PWVct to reliably infer central arterial stiffness in older populations.

Clinically, central PWVcf is a well-established biomarker for cardiovascular risk, strongly linked to adverse outcomes through its assessment of central arterial stiffness. While PWVct offers a non-occlusive and accessible alternative, its reduced sensitivity to age-related changes limits its value as a direct surrogate for PWVcf, especially in older individuals. Future use of PWVct should acknowledge these limitations and consider it a complementary measure rather than a replacement for central PWV. Further studies are needed to refine PWVct methodologies, improve its alignment with PWVcf, and evaluate its clinical relevance across diverse populations.

### 5.2 Agreement between PCG-based PWV and reference PWVcf devices

PWVcf measurements from PCG-based methods were broadly comparable to those from historical datasets, with no significant differences observed in the overall analysis. However, interaction effects between age and study type suggested subtle differences in how devices measure age-related changes. These effects diminished when historical data were resampled to match our study size, indicating that differences were influenced by unequal sample sizes.

Device-specific comparisons revealed baseline differences between PCG-based PWVcf and reference devices, including SphygmoCor, Vicorder, and Pulse Pen, but not Complior. Interaction effects between age and study type were also observed for most devices, reflecting variability in how different devices capture the age-PWVcf relationship. After resampling, baseline differences for most devices were no longer evident, except for Pulse Pen, which continued to show significant differences. Among the resampled devices, Vicorder exhibited a more pronounced interaction effect, suggesting it may differ more substantially in its age-PWVcf measurements compared to PCG-based methods.

The observed discrepancies between devices likely arise from variations in methodology and calibration protocols. For instance, the SphygmoCor uses applanation tonometry at the carotid artery and a pressure cuff at the femoral artery to calculate pulse transit time via the foot-to-foot method (44–49). This method requires skilled operators to ensure accurate arterial compression and waveform acquisition. In contrast, the Vicorder uses oscillometric cuffs at the carotid and femoral sites to capture pressure waveforms (48–51). While this approach is non-invasive and operator-friendly, it may smooth finer details of arterial waveforms, particularly in older individuals with increased arterial stiffening. The Pulse Pen also employs tonometry but captures waveforms sequentially from the carotid and femoral arteries, which, combined with its reliance on proprietary algorithms for waveform processing and operator technique for arterial site compression, may introduce additional variability (52). The Complior, which uses automated pressure waveform analysis with piezoelectric sensors at arterial sites, reduces operator dependency and incorporates a standardized distance calculation in its software, using a factor of 0.8 to estimate arterial distance (34, 53, 54). This standardization may explain its closer alignment with PCG-based measurements.

The observed differences between devices may be due to the proprietary algorithms and signal filtering techniques used to calculate pulse transit time, which can introduce variability across methods. Additionally, differences in waveform acquisition—whether from tonometry, PPG, or oscillometric cuffs—may capture distinct features of the arterial waveform, further amplifying these variations. In contrast, our PPG-based system avoids hidden filters or proprietary algorithms, using automated ECG peak-to-pressure waveform foot timing to determine transit delays. These findings underscore the need for standardized methodologies and algorithms across devices to enhance inter-device comparability and reliability in future studies.

### 5.3 Distensibility index vs. age and relationship with PWV

In Substudy #1, we assessed arterial distensibility indices at the aortic and carotid arteries. Significant correlations were found between PWV and aortic distensibility index (AAo Dis), but not carotid distensibility index (CCA Dis). Specifically, AAo Dis showed a negative correlation with both PWVcf PWVct. This is consistent with the understanding that PWV is a measure of aortic or central arterial stiffness. The lack of correlation with CCA Dis suggests that local carotid and aortic mechanics are distinct and cannot be used interchangeably. Age was also a significant predictor of AAo Dis, further supporting the role of aging in reducing central arterial elasticity. This finding emphasizes that as individuals age, their aortic arteries become less elastic, contributing to increased arterial stiffness (73).

### 5.4 Test–retest

Both the semi-occlusive and non-occlusive PWV methods displayed good repeatability. The reliability of PCG in detecting pulse wave travel delays at various anatomical sites, coupled with the reproducibility and repeatability of our setup, makes it a viable alternative to current more expensive devices. These reproducibility results are comparable to existing commercial devices like Complior and PulsePen, which are considered dependable for clinical use (31, 63, 71).

### 5.5 Enhanced comfort, accessibility and reduced costs

Our findings demonstrate that the PCG-based system offers significant advantages over traditional tonometric devices. Traditional devices like SphygmoCor and Vicorder are expensive and require specialized training, limiting their use in routine clinical practice and resource-limited settings. Additionally, tonometry can be intrusive and uncomfortable, especially when assessed at the femoral arterial site. Some groups have even suggested that the applied pressure from tonometry may influence the measurement (35, 36). We did not observe this in our small substudy although there was non-statistically significant trend. In contrast, our system's low cost, comfort, and ease of use could facilitate more accessible and widespread screening for arterial stiffness and related cardiovascular risks.

### 5.6 Continuous monitoring potential

The continuous nature of our PWV measurements offers the potential for real-time monitoring of arterial stiffness, which could provide additional insights into vascular health (74). While this capability might help detect transient changes in arterial stiffness that could be missed by intermittent measurements, the clinical utility of continuous PWV monitoring remains uncertain. Confounding factors can influence short-term PWV variations, making it challenging to apply in practice. Further research is needed to explore its potential benefits in managing patients with fluctuating cardiovascular conditions or during acute physiological changes.

### 5.7 Capturing peripheral arterial stiffness

There may also be additional value in combined PWVcf and PWVct measurements, which is feasible with our approach. A Bland–Altman analysis comparing PWVcf and PWVct revealed an increasing bias with age, reflecting the marked increase in central arterial stiffness relative to the more stable stiffness of peripheral arteries. This indicates that PWVct may be a less reliable surrogate for central PWV in older populations. However, recent studies have highlighted the importance of assessing peripheral arterial stiffness alongside aortic stiffness (55–57). Therefore, our system's ability to also capture peripheral arterial stiffness changes may provide important insights into arterial remodeling and its relationship with age or disease.

### 5.8 Clinical integration

The PCG-based system's non-invasive design, affordability, and ease of use hold promise for eventual integration into clinical workflows. In primary care, it could serve as a convenient screening tool for arterial stiffness during routine visits, offering both precise central stiffness measurements (PWVcf) and a quick, comfortable option (PWVct) for broader population assessments. However, additional validation is necessary to ensure its reliability and effectiveness.

In specialty clinics (e.g., hypertension or diabetes management), the system may facilitate regular tracking of arterial stiffness, helping clinicians detect early changes and initiate timely interventions such as lifestyle modifications or pharmacotherapies (75). Its ability to measure both central and peripheral stiffness also provides more comprehensive monitoring of arterial remodeling over time. Nonetheless, further research is warranted to confirm its clinical utility across a wider range of patient populations.

Finally, incorporating PWV into existing cardiovascular risk models (e.g., Framingham Risk Score, ASCVD risk) could improve risk stratification (76). By combining standard metrics (e.g., blood pressure and cholesterol) with PWV data, clinicians may gain deeper insights into a patient's cardiovascular risk profile (77, 78). Future studies should focus on validating the PCG-based PWV approach in diverse populations and determining its impact on clinical decision-making and outcomes.

### 5.9 Future research implications and limitations

Our approach reduces the barriers to conducting large-scale studies on arterial stiffness. By eliminating the need for expensive and complex equipment, our system enables more researchers to participate in this field of study. This democratization of technology may accelerate the pace of discovery and improve our understanding of cardiovascular health across diverse populations.

One significant limitation of our study is the small sample size of 63 participants, with a predominance of female participants (78%). This imbalance may introduce bias, as arterial stiffness parameters can differ between sexes due to physiological and hormonal differences (79, 80) This bias could limit the generalizability of our findings to broader populations, particularly male cohorts. Future studies should involve larger and more demographically diverse populations to validate the findings and assess the reproducibility of the PCG-based system across various subgroups.

Additionally, we did not validate PWV against commercially available devices (31). Therefore, we are unable to assess how accurate and precise our methods are on the individual level. Additionally, our study primarily involved healthy volunteers, excluding those with hypertension and other conditions. Further studies should explore the agreement of PCG-based approaches in patient populations with known disease.

We also recognize that our non-occlusive PWVct method incorporates peripheral arteries, which may introduce variability, particularly in older populations where peripheral arterial stiffness increases. The observed bias in PWVct with increasing age suggests the need for caution when interpreting peripheral PWV as a surrogate for central PWV in older adults. This limitation highlights that while PWVct offers a non-occlusive, low-cost alternative, it may be less dependable in aging populations.

Moreover, our study used brachial blood pressure as a surrogate for central pulse pressure in calculating the inverted stiffness index. While this approach is non-invasive and commonly used, it introduces potential bias due to pulse pressure amplification effects. Pulse pressure amplification decreases with age, leading to different biases in distensibility calculations depending on the age group. In younger subjects, non-invasive brachial blood pressure tends to overestimate true diastolic pressure in the abdominal aorta (72). Additionally, vessel area measurements were performed using manual edge detection, which is less reliable than automated wall detection algorithms. This introduces variability and may impact the accuracy of the distensibility and stiffness indices, particularly when assessing central arterial stiffness. These methodological limitations should be considered when interpreting our findings.

An additional consideration is the accuracy of PWV measurements when performed by medical staff with limited methodological expertise. Although our device is designed to be easy to use, there is a risk of potential errors if the operator is unaware of sources of error or confounding factors. Future research should focus on streamlining the use of these devices for inexperienced operators, validating cuffless and semi-cuffless approaches in diverse and clinical populations, and improving patient comfort to enhance usability.

Despite these limitations, the strengths of our system—cost-effectiveness, ease of use, and comfort—position it as a valuable complementary tool to established tonometric methods to assess PWV.

### 5.10 Summary and importance

The availability of a low-cost, non-occlusive PWV measurement system represents a major step forward in the field of cardiovascular health monitoring. By making this technology accessible to a wider population, we can enhance screening and monitoring capabilities, leading to better health outcomes.

## Data Availability

The raw data supporting the conclusions of this article will be made available by the authors, without undue reservation.

## References

[1] Bonfioli GB Rodella L Rosati R Carrozza A Metra M Vizzardi E. Aortopathies: from etiology to the role of arterial stiffness. J Clin Med. (2023) 12:3949. 10.3390/JCM12123949 37373642 PMC10298905

[2] Shirwany NA Zou MH. Arterial stiffness: a brief review. Acta Pharmacol Sin. (2010) 31(10):1267. 10.1038/APS.2010.123 20802505 PMC3078647

[3] Laurent S Boutouyrie P. Arterial stiffness and hypertension in the elderly. Front Cardiovasc Med. (2020) 7:544302. 10.3389/FCVM.2020.544302/BIBTEX 33330638 PMC7673379

[4] González LD Romero-Orjuela SP Rabeya FJ del Castillo V Echeverri D. Age and vascular aging: an unexplored frontier. Front Cardiovasc Med. (2023) 10:1278795. 10.3389/FCVM.2023.1278795/BIBTEX 38028481 PMC10665864

[5] Tucker WD Arora Y Mahajan K. Anatomy, Blood Vessels. Treasure Island, FL: StatPearls Publishing (2023). Available online at: https://www.ncbi.nlm.nih.gov/books/NBK470401/ (accessed June 5, 2024). 29262226

[6] Vlachopoulos C Aznaouridis K Stefanadis C. Prediction of cardiovascular events and all-cause mortality with arterial stiffness: a systematic review and meta-analysis. J Am Coll Cardiol. (2010) 55(13):1318–27. 10.1016/J.JACC.2009.10.061 20338492

[7] Bonarjee VVS. Arterial stiffness: a prognostic marker in coronary heart disease. Available methods and clinical application. Front Cardiovasc Med. (2018) 5:64. 10.3389/FCVM.2018.00064 29951487 PMC6008540

[8] Boutouyrie P Tropeano AI Asmar R Gautier I Benetos A Lacolley P Aortic stiffness is an independent predictor of primary coronary events in hypertensive patients. Hypertension. (2002) 39(1):10–5. 10.1161/HY0102.099031 11799071

[9] Regnault V Lacolley P Laurent S. Arterial stiffness: from basic primers to integrative physiology. Annu Rev Physiol. (2024) 86:99–121. 10.1146/ANNUREV-PHYSIOL-042022-031925/CITE/REFWORKS 38345905

[10] Reeve EH Barnes JN Moir ME Walker AE. Impact of arterial stiffness on cerebrovascular function: a review of evidence from humans and preclinical models. Am J Physiol Heart Circ Physiol. (2024) 326(3):H689–704. 10.1152/AJPHEART.00592.2023 38214904 PMC11221809

[11] Heffernan KS Stoner L London AS Augustine JA Lefferts WK. Estimated pulse wave velocity as a measure of vascular aging. PLoS One. (2023) 18(1):e0280896. 10.1371/JOURNAL.PONE.0280896 36701358 PMC9879446

[12] Pai RG Shah PM. Relationship between the pulse wave and the flow velocity wave and their propagation velocities in the arterial system: implications for the assessment of regional physical properties of the arterial beds. Int J Angiol. (1999) 8(2):127–30. 10.1007/BF01616831/METRICS

[13] Callaghan FJ Geddes LA Babbs CF Bourland JD. Relationship between pulse-wave velocity and arterial elasticity. Med Biol Eng Comput. (1986) 24(3):248–54. 10.1007/BF02441620/METRICS 3747623

[14] Posey JA Geddes LA. Measurement of the modulus of elasticity of the arterial wall. (1973). Available online at: https://scholars.houstonmethodist.org/en/publications/measurement-of-the-modulus-of-elasticity-of-the-arterial-wall (accessed December 12, 2024).

[15] Korteweg D. Ber die fortpflanzungsgeschwindigkeit des schalles in elastischen rohren. Ann Phys Chem Neue Folge. (1878) 5:525–42. Available online at: https://cir.nii.ac.jp/crid/1570854174308525568.bib?lang=en 10.1002/andp.18782411206

[16] Newman DL Greenwald SE. Validity of the Moens-Korteweg equation. In: Bauer RD Busse R, editors. The Arterial System. Berlin, Heidelberg: Springer (1978). 10.1007/978-3-642-67020-6_10

[17] Wentland AL Grist TM Wieben O. Review of MRI-based measurements of pulse wave velocity: a biomarker of arterial stiffness. Cardiovasc Diagn Ther. (2014) 4(2):193. 10.3978/J.ISSN.2223-3652.2014.03.04 24834415 PMC3996237

[18] Ohyama Y Ambale-Venkatesh B Noda C Kim JY Tanami Y Teixido-Tura G Aortic arch pulse wave velocity assessed by magnetic resonance imaging as a predictor of incident cardiovascular events: the MESA (multi-ethnic study of atherosclerosis). Hypertension. (2017) 70(3):524–30. 10.1161/HYPERTENSIONAHA.116.08749/-/DC1 28674039 PMC5612667

[19] Millasseau SC Stewart AD Patel SJ Redwood SR Chowienczyk PJ. Evaluation of carotid-femoral pulse wave velocity. Hypertension. (2005) 45(2):222–6. 10.1161/01.HYP.0000154229.97341.D2 15642772

[20] Laurent S Cockcroft J Van Bortel L Boutouyrie P Giannattasio C Hayoz D Expert consensus document on arterial stiffness: methodological issues and clinical applications. Eur Heart J. (2006) 27(21):2588–605. 10.1093/eurheartj/ehl254 17000623

[21] Dogui A Kachenoura N Frouin F Lefort M De Cesare A Mousseaux E Consistency of aortic distensibility and pulse wave velocity estimates with respect to the bramwell-hill theoretical model: a cardiovascular magnetic resonance study. J Cardiovasc Magn Reson. (2011) 13(1):11. 10.1186/1532-429X-13-11 21272312 PMC3038969

[22] Salvi P Valbusa F Kearney-Schwartz A Labat C Grillo A Parati G Non-invasive assessment of arterial stiffness: pulse wave velocity, pulse wave analysis and carotid cross-sectional distensibility: comparison between methods. J Clin Med. (2022) 11(8):2225. 10.3390/JCM11082225 35456316 PMC9029786

[23] Nelson MR Stepanek J Cevette M Covalciuc M Hurst RT Tajik AJ. Noninvasive measurement of central vascular pressures with arterial tonometry: clinical revival of the pulse pressure waveform? Mayo Clin Proc. (2010) 85(5):460. 10.4065/MCP.2009.0336 20435839 PMC2861976

[24] Jiang B Liu B McNeill KL Chowienczyk PJ. Measurement of pulse wave velocity using pulse wave Doppler ultrasound: comparison with arterial tonometry. Ultrasound Med Biol. (2008) 34(3):509–12. 10.1016/J.ULTRASMEDBIO.2007.09.008 18031922

[25] Butlin M Qasem A Battista F Bozec E McEniery CM Millet-Amaury E Carotid-femoral pulse wave velocity assessment using novel cuff-based techniques: comparison with tonometric measurement. J Hypertens. (2013) 31(11):2237–43. 10.1097/HJH.0B013E328363C789 24077246

[26] Tomiyama H Yamashina A Arai T Hirose K Koji Y Chikamori T Influences of age and gender on results of noninvasive brachial–ankle pulse wave velocity measurement—a survey of 12517 subjects. Atherosclerosis. (2003) 166(2):303–9. 10.1016/S0021-9150(02)00332-5 12535743

[27] Alivon M Phuong TVD Vignon V Bozec E Khettab H Hanon O A novel device for measuring arterial stiffness using finger-toe pulse wave velocity: validation study of the pOpmètre®. Arch Cardiovasc Dis. (2015) 108(4):227–34. 10.1016/J.ACVD.2014.12.003 25682547

[28] Chen YS Lu WA Hsu LY Kuo CD. Determinants of hand pulse wave velocity and hand pulse transit time in healthy adults. Sci Rep. (2024) 14(1):1–7. 10.1038/s41598-024-60927-5 38698185 PMC11066034

[29] Sugawara J Hayashi K Yokoi T Cortez-Cooper MY DeVan AE Anton MA Brachial–Ankle pulse wave velocity: an index of central arterial stiffness? J Hum Hypertens. (2005) 19(5):401–6. 10.1038/sj.jhh.1001838 15729378

[30] Segers P Kips J Trachet B Swillens A Vermeersch S Mahieu D Limitations and pitfalls of non-invasive measurement of arterial pressure wave reflections and pulse wave velocity. Artery Res. (2009) 3(2):79–88. 10.1016/J.ARTRES.2009.02.006

[31] Spronck B Terentes-Printzios D Avolio AP Boutouyrie P Guala A Jerončić A 2024 recommendations for validation of noninvasive arterial pulse wave velocity measurement devices. Hypertension. (2023) 81(1):183–92. 10.1161/HYPERTENSIONAHA.123.21618 37975229 PMC10734786

[32] Salvi P. Pulse waves (2012).

[33] Milan A Zocaro G Leone D Tosello F Buraioli I Schiavone D Current assessment of pulse wave velocity: comprehensive review of validation studies. J Hypertens. (2019) 37(8):1547–57. 10.1097/HJH.0000000000002081 30882597

[34] van Velzen MHN Stolker RJ Loeve AJ Niehof SP Mik EG. Comparison between pulse wave velocities measured using complior and measured using biopac. J Clin Monit Comput. (2019) 33(2):241–7. 10.1007/S10877-018-0165-9/TABLES/3 29876710 PMC6420418

[35] Jobbágy Á Nagy P. The effect of occlusion with the cuff. IFMBE Proc. (2018) 65:9–12. 10.1007/978-981-10-5122-7_3

[36] Tanaka H Mito A Hirano H Soh Z Nakamura R Saeki N Estimation of arterial viscosity based on an oscillometric method and its application in evaluating the vascular endothelial function. Sci Rep. (2019) 9(1):1–11. 10.1038/s41598-019-38776-4 30796239 PMC6384877

[37] Butlin M Qasem A. Large artery stiffness assessment using SphygmoCor technology. Pulse. (2017) 4(4):180. 10.1159/000452448 28229053 PMC5290450

[38] Liu AB Hsu PC Chen ZL Wu HT. Measuring pulse wave velocity using ECG and photoplethysmography. J Med Syst. (2011) 35(5):771–7. 10.1007/S10916-010-9469-0/FIGURES/5 20703725

[39] Loukogeorgakis S Dawson R Phillips N Martyn CN Greenwald SE. Validation of a device to measure arterial pulse wave velocity by a photoplethysmographic method. Physiol Meas. (2002) 23(3):581–96. 10.1088/0967-3334/23/3/309 12214765

[40] Janjua GMW Finlay D Guldenring D Ul Haq A McLaughlin J. A low-cost tonometer alternative: a comparison between photoplethysmogram and finger ballistocardiogram and validation against tonometric waveform. IEEE Access. (2019) 7:142787–95. 10.1109/ACCESS.2019.2944212

[41] Elgendi M Fletcher R Liang Y Howard N Lovell NH Abbott D The use of photoplethysmography for assessing hypertension. NPJ Digit Med. (2019) 2(1):1–11. 10.1038/s41746-019-0136-7 31388564 PMC6594942

[42] Kusche R Klimach P Malhotra A Kaufmann S Ryschka M. An in-ear pulse wave velocity measurement system using heart sounds as time reference. Curr Dir Biomed Eng. (2015) 1(1):366–70. 10.1515/CDBME-2015-0090/MACHINEREADABLECITATION/RIS

[43] Kusche R Lindenberg AV Hauschild S Ryschka M. Aortic pulse wave velocity measurement via heart sounds and impedance plethysmography. IFMBE Proc. (2018) 68(2):843–6. 10.1007/978-981-10-9038-7_155/FIGURES/4

[44] Jelinek M Dobeš J Poušek L Hána K. Using a phonocardiography in a pulse wave velocity measurement. Proceedings of the 3rd IEEE International Symposium on Signal Processing and Information Technology, ISSPIT 2003 (2003). p. 491–3. 10.1109/ISSPIT.2003.1341165

[45] Gaddum NR Alastruey J Beerbaum P Chowienczyk P Schaeffter T. A technical assessment of pulse wave velocity algorithms applied to non-invasive arterial waveforms. Ann Biomed Eng. (2013) 41(12):2617–29. 10.1007/S10439-013-0854-Y/FIGURES/6 23817766

[46] Feitosa ADM Mota-Gomes MA Barroso WS Miranda RD Barbosa ECD Brandão AA The impact of changing home blood pressure monitoring cutoff from 135/85 to 130/80 mmHg on hypertension phenotypes. J Clin Hypertens. (2021) 23(7):1447. 10.1111/JCH.14261 PMC8678775 33955645

[47] Davis LL. Hypertension: how low to go when treating older adults. J Nurse Pract. (2019) 15(1):1–6. 10.1016/j.nurpra.2018.10.010 31354390 PMC6660175

[48] Parikh JD Hollingsworth KG Kunadian V Blamire A MacGowan GA. Measurement of pulse wave velocity in normal ageing: comparison of vicorder and magnetic resonance phase contrast imaging. BMC Cardiovasc Disord. (2016) 16(1):1–7. 10.1186/S12872-016-0224-4/TABLES/6 26892669 PMC4759948

[49] Müller J Oberhoffer R Barta C Hulpke-Wette M Hager A. Oscillometric carotid to femoral pulse wave velocity estimated with the vicorder device. J Clin Hypertens (Greenwich). (2013) 15(3):176–9. 10.1111/JCH.12045 23458589 PMC8033822

[50] Bailey MA Davies JM Griffin KJ Bridge KI Johnson AB Sohrabi S Carotid-femoral pulse wave velocity is negatively correlated with aortic diameter. Hypertens Res. (2014) 37(10):926–32. 10.1038/hr.2014.101 24919482

[51] Sultan SR. The association of arterial pulse wave velocity with internal carotid artery blood flow in healthy subjects: a pilot study. Artery Res. (2024) 30(1):1–8. 10.1007/S44200-024-00053-9/FIGURES/339086596

[52] Salvi P Lio G Labat C Ricci E Pannier B Benetos A. Validation of a new non-invasive portable tonometer for determining arterial pressure wave and pulse wave velocity: the PulsePen device. J Hypertens. (2004) 22(12):2285–93. 10.1097/00004872-200412000-00010 15614022

[53] Kozakova M Morizzo C Guarino D Federico G Miccoli M Giannattasio C The impact of age and risk factors on carotid and carotid-femoral pulse wave velocity. J Hypertens. (2015) 33(7):1446–51. 10.1097/HJH.0000000000000582 26039533

[54] Magalhães P Capingana DP Silva ABT Ferreira AVL De Sá Cunha R Rodrigues SL Age- and gender-specific reference values of pulse wave velocity for African adults: preliminary results. Age (Omaha). (2013) 35(6):2345. 10.1007/S11357-012-9504-9 PMC3824996 23319362

[55] Plotdigitizer. PlotDigitizer Online App. Available online at: https://plotdigitizer.com/app (accessed July 8, 2024).

[56] Morioka T Mori K Emoto M. Is stiffness parameter *β* useful for the evaluation of atherosclerosis? Its clinical implications, limitations, and future perspectives. J Atheroscler Thromb. (2021) 28(5):435. 10.5551/JAT.RV17047 33583910 PMC8193788

[57] Godia EC Madhok R Pittman J Trocio S Ramas R Cabral D Carotid artery distensibility. J Ultrasound Med. (2007) 26(9):1157–65. 10.7863/JUM.2007.26.9.1157 17715309 PMC2677175

[58] Nguyen T Nguyen T-TA Powell EN Chang A Bush A. Rapid, low-cost measurement of carotid artery distensibility in children with sickle cell disease using compact point-of-care ultrasound. Blood. (2023) 142(Suppl 1):5329. 10.1182/BLOOD-2023-190185

[59] Hayashi K Handa H Nagasawa S Okumura A Moritake K. Stiffness and elastic behavior of human intracranial and extracranial arteries. J Biomech. (1980) 13(2):175–84. 10.1016/0021-9290(80)90191-8 7364778

[60] Hashim A Tahir MJ Ullah I Asghar MS Siddiqi H Yousaf Z. The utility of point of care ultrasonography (POCUS). Ann Med Surgery. (2021) 71:102982. 10.1016/J.AMSU.2021.102982 PMC8606703 34840746

[61] Haapala EA Veijalainen A Kujala UM Finni T. Reproducibility of pulse wave velocity and augmentation index derived from non-invasive occlusive oscillometric tonometry analysis in adolescents. Clin Physiol Funct Imaging. (2019) 39(1):22–8. 10.1111/CPF.12528 29862639

[62] Miyatani M Masani K Moore C Szeto M Oh P Craven C. Test–retest reliability of pulse wave velocity in individuals with chronic spinal cord injury. J Spinal Cord Med. (2012) 35(5):400. 10.1179/2045772312Y.0000000042 23031177 PMC3459569

[63] Podrug M Šunjić B Koren P Đogaš V Mudnić I Boban M What is the smallest change in pulse wave velocity measurements that can be attributed to clinical changes in arterial stiffness with certainty: a randomized cross-over study. J Cardiovasc Dev Dis. (2023) 10(2):44. 10.3390/JCDD10020044 36826540 PMC9962359

[64] Forthofer RN Lee ES Hernandez M. 4 - probability and life tables. In Biostatistics. 2nd Edn. Burlington, MA: Academic Press (2007). p. 71–102. 10.1016/B978-0-12-369492-8.50009-1

[65] McEniery CM Yasmin N Hall IR Qasem A Wilkinson IB Cockcroft JR. Normal vascular aging: differential effects on wave reflection and aortic pulse wave velocity: the anglo-cardiff collaborative trial (ACCT). J Am Coll Cardiol. (2005) 46(9):1753–60. 10.1016/J.JACC.2005.07.037 16256881

[66] Stabouli S Printza N Zervas C Dotis J Chrysaidou K Maliahova O Comparison of the SphygmoCor XCEL device with applanation tonometry for pulse wave velocity and central blood pressure assessment in youth. J Hypertens. (2019) 37(1):30–6. 10.1097/HJH.0000000000001819 29939943

[67] Vriz O Driussi C Carrubba S La Bello V Di Zito C Carerj S Comparison of sequentially measured aloka echo-tracking one-point pulse wave velocity with SphygmoCor carotid–femoral pulse wave velocity. SAGE Open Med. (2013) 1:205031211350756. 10.1177/2050312113507563 PMC4687782 26770685

[68] Hickson SS Butlin M Broad J Avolio AP Wilkinson IB McEniery CM. Validity and repeatability of the vicorder apparatus: a comparison with the SphygmoCor device. Hypertens Res. (2009) 32(12):1079–85. 10.1038/HR.2009.154 19779487

[69] Elias M Dore GA Davey A Abhayaratna W Goodell AL Robbins M. Norms and reference values for pulse wave velocity: one size does not fit all. J Biosci Med. (2011) 1(1):1–10. 10.5780/JBM2011.4

[70] Shiburi CP Staessen JA Maseko M Wojciechowska W Thijs L Van Bortel LM Reference values for SphygmoCor measurements in South Africans of African ancestry. Am J Hypertens. (2006) 19(1):40–6. 10.1016/J.AMJHYPER.2005.06.018/2/AJH.40.F3.JPEG 16461189

[71] Grillo A Parati G Rovina M Moretti F Salvi L Gao L Short-term repeatability of noninvasive aortic pulse wave velocity assessment: comparison between methods and devices. Am J Hypertens. (2018) 31(1):80–8. 10.1093/AJH/HPX140 29059329

[72] Mattace-Raso FUS Hofman A Verwoert GC Wittemana JCM Wilkinson I Cockcroft J Determinants of pulse wave velocity in healthy people and in the presence of cardiovascular risk factors: ‘establishing normal and reference values’. Eur Heart J. (2010) 31(19):2338–50. 10.1093/EURHEARTJ/EHQ165 20530030 PMC2948201

[73] Mammoto A Matus K Mammoto T. Extracellular matrix in aging aorta. Front Cell Dev Biol. (2022) 10:822561. 10.3389/FCELL.2022.822561/BIBTEX 35265616 PMC8898904

[74] Yi Z Liu Z Li W Ruan T Chen X Liu J Piezoelectric dynamics of arterial pulse for wearable continuous blood pressure monitoring. Adv Mater. (2022) 34(16):2110291. 10.1002/ADMA.202110291 35285098

[75] Pilz N Heinz V Ax T Fesseler L Patzak A Bothe TL. Pulse wave velocity: methodology, clinical applications, and interplay with heart rate variability. Rev Cardiovasc Med. (2024) 25(7):266. 10.31083/J.RCM2507266 39139426 PMC11317333

[76] Chang G Hu Y Ge Q Chu S Avolio A Zuo J. Arterial stiffness as a predictor of the Index of atherosclerotic cardiovascular disease in hypertensive patients. Int J Environ Res Public Health. (2023) 20(4):2832. 10.3390/IJERPH20042832 36833532 PMC9957494

[77] Mancia G Kreutz R Brunstr M Burnier M Grassi G Januszewicz A ESH Guidelines (2023). Available online at: www.jhypertension.com (accessed January 3, 2025).

[78] Visseren F Mach F Smulders YM Carballo D Koskinas KC Bäck M 2021 ESC guidelines on cardiovascular disease prevention in clinical practice. Eur Heart J. (2021) 42(34):3227–337. 10.1093/EURHEARTJ/EHAB484 34458905

[79] van Hout MJ Dekkers IA Westenberg JJ Schalij MJ Widya RL de Mutsert R Normal and reference values for cardiovascular magnetic resonance-based pulse wave velocity in the middle-aged general population. J Cardiovasc Magn Reson. (2021) 23(1):1–10. 10.1186/S12968-021-00739-Y/FIGURES/4 33866975 PMC8054386

[80] Kehmeier MN Walker AE. Sex differences in large artery stiffness: implications for cerebrovascular dysfunction and Alzheimer’s disease. Front Aging. (2021) 2:791208. 10.3389/FRAGI.2021.791208/BIBTEX 35072153 PMC8782423

