# Supplementary material for: Phonocardiography based pulse wave velocity system for non-occlusive assessment of arterial stiffness
Source: Front Cardiovasc Med. 2025 Jan 23;12:1481836. doi: 10.3389/fcvm.2025.1481836 (PMC11798789; doi:10.3389/fcvm.2025.1481836)
Supplement: Supplementary file 1 [file Datasheet1.docx]

**Phonocardiography Based Pulse Wave Velocity System for Non-Occlusive Assessment of Arterial Stiffness:**

**Supplementary Material**

When comparing TTF delays (in seconds) across measurement types and sites we found delays of: 0.036 ± 0.015 for PCG at the heart, 0.084 ± 0.019 for the carotid, 0.184 ± 0.039 for PCG at the unoccluded femoral artery, 0.223 ± 0.069 for PCG at the occluded femoral artery, 0.233 ±0.034 for the pressure cuff at the femoral artery, and 0.003 ± 0.0002 for the calculated speed of sound delay from the heart to the femoral artery (**Supplementary Figure 1**).

Mean differences in PCG time-to-foot (TTF) delay from the ECG R-peak to various anatomical locations distal from the heart showed statistically significant delays from a Tukey Test comparing all paired combinations of means. TTF delays from all femoral locations (PCG and cuffs) were not statistically different (p>0.05), while they were all statistically different (p<0.05) from soft tissue sound travel speed at the femoral artery (**Supplementary Figure 1**).


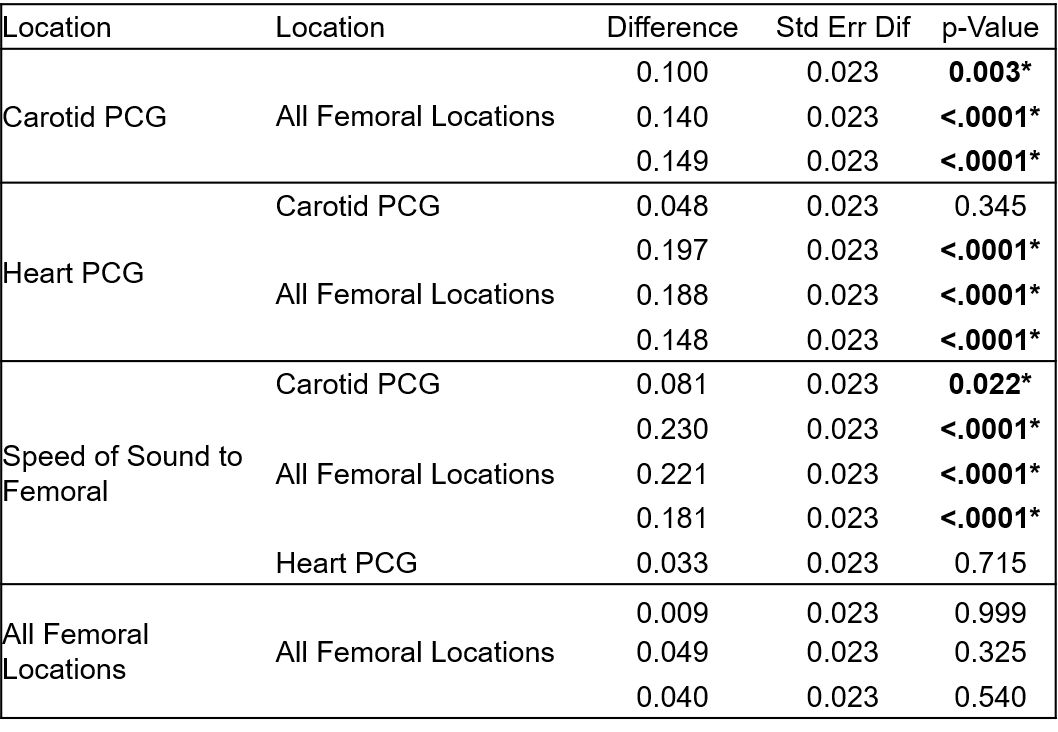


Tukey HSD Pairwise Differences Report

**Supplementary Figure 1**: Mean differences in PCG time-to-foot (TTF) delay from ECG R-peak to various anatomical locations distal from the heart. Showing statistically significant delays (red) from a Tukey Test pairwise comparison of means. TTF delays from all femoral locations (PCG and cuffs) were not statistically different (p>0.05), while they were all statistically different (p<0.05) from the speed of sound at the femoral.
